# Supplementary material for: Genome Size of Life Forms of Araceae—A New Piece in the C-Value Puzzle
Source: Plants (Basel). 2022 Jan 27;11(3):334. doi: 10.3390/plants11030334 (PMC8840116; doi:10.3390/plants11030334)
Supplement: Supplementary file 1 [file plants-11-00334-s001.zip › plants-1553320-supplementary.pdf]

plants MDPI

Supplement for the Article

Genome Size of Life Forms of *Araceae*—A New Piece in the C-Value Puzzle

Domen Kocjan [1,†], Jasna Dolenc Koce [2,†,*], Florian Etl [3], Marina Dermastia [4]

Table S1. 2C-values and life forms of aroid species used in the present analysis, taken from the Royal Botanic Gardens Kew database [14]. When two different authors measured the same species, both 2C values are included, resulting in duplicate values for some species.

Genus	Species	Life form	2C [pg DNA]
Alocasia	*A. cucullata*	Terrestrial	16.74
	A. longiloba	Terrestrial	18.70
	A. reginula	Terrestrial	9.90
	A. sanderiana	Terrestrial	10.10
	A. sp.	Terrestrial	20.30
	A. zebrina var. tigrina cv. Superba	Terrestrial	11.20
Amorphophallus	*A. abyssinicus*	Terrestrial	21.50
	A. bulbifer	Terrestrial	18.60
	A. commutatus	Terrestrial	23.50
	A. gallaensis	Terrestrial	23.50
	A. goetzei	Terrestrial	22.60
	A. hildebrandtii	Terrestrial	25.60
	A. johnsonii	Terrestrial	31.70
	A. konjac	Terrestrial	12.95
	A. krausei	Terrestrial	12.30
	A. lambii	Terrestrial	15.30
	A. lewallei	Terrestrial	27.80
	A. oncophyllus	Terrestrial	19.40
	A. paeoniifolius	Terrestrial	8.40
	A. prainii	Terrestrial	7.50
Anthurium	*A. amnicola*	Aquatic (rheophyte)	10.36
	A. andraeanum	Facultative epiphyte	9.59
	A. antioquiense	Aquatic (rheophyte)	9.83
	A. armeniense	Obligatory epiphyte	12.01
	A. bakeri	Obligatory epiphyte	9.28
	A. cerrocampanense	Facultative epiphyte	11.44
	A. clavigerum	Obligatory epiphyte	14.27
	A. clidemioides	Obligatory epiphyte	9.17
	A. coriaceum	Facultative epiphyte	14.32

	A. esmeraldense	Facultative epiphyte	8.79
	A. flexile subsp. *muelleri*	Obligatory epiphyte	9.46
	A. formosum	Facultative epiphyte	8.77
	A. fragrantissimum	Obligatory epiphyte	6.21
	A. gracile	Obligatory epiphyte	12.38
	A. grande	Facultative epiphyte	27.05
	A. gymnopus	Hemiepiphyte	11.21
	A. hoffmanni	Facultative epiphyte	9.72
	A. hookeri	Facultative epiphyte	8.07
	A. kamemotoanum	Facultative epiphyte	9.47
	A. lentii	Facultative epiphyte	13.96
	A. leuconeurum	Terrestrial	14.35
	A. lucens	Facultative epiphyte	13.04
	A. microspadix	Facultative epiphyte	12.25
	A. nymphaeifolium	Facultative epiphyte	9.45
	A. obtusum	Obligatory epiphyte	5.11
	A. ochranthum	Terrestrial	10.75
	A. pittieri	Obligatory epiphyte	5.61
	A. radicans	Terrestrial	14.36
	A. ravenii	Obligatory epiphyte	10.43
	A. roseospadix	Facultative epiphyte	20.83
	A. scandens subsp. *pusillum*	Obligatory epiphyte	5.12
	A. scandens subsp. *scandens*	Obligatory epiphyte	9.64
	A. schlechtendalii subsp. *schlechtendalii*	Obligatory epiphyte	12.55
	A. solitarium	Obligatory epiphyte	15.36
	A. warocqueanum	Obligatory epiphyte	8.86
	A. watermaliense	Facultative epiphyte	8.52
	A. wendlingeri	Obligatory epiphyte	7.45
Anubias	*A. barteri* var. nana	Aquatic (rheophyte)	4.10
Arisaema	*A. ciliatum*	Terrestrial	9.78
	A. dracontium	Terrestrial	6.90
	A. flavum	Terrestrial	10.12
	A. triphyllum	Terrestrial	18.40
Arisarum	*A. proboscidium*	Terrestrial	17.90
	A. simorrhinum	Terrestrial	29.89
	A. vulgare	Terrestrial	29.12
Arum	*A. concinnatum*	Terrestrial	23.92
	A. cylindraceum	Terrestrial	9.05
	A. dioscoridis	Terrestrial	21.17
	A. hygrophilum	Terrestrial	20.86
	A. italicum	Terrestrial	25.27
	A. maculatum	Terrestrial	21.80
	A. orientale	Terrestrial	8.92

Biarum	*B. olivieri*	Terrestrial	6.82
	B. tenuifolium	Terrestrial	6.20
Caladium	*C. bicolor* var. red polka large	Terrestrial	20.26
	C. bicolor var. red polka	Terrestrial	11.04
	C. praetermissum	Terrestrial	7.51
Calla	*C. palustris*	Aquatic (helophyte)	2.16
Colocasia	*C. esculenta*	Terrestrial	6.60
	C. esculenta	Terrestrial	8.10
Cryptocoryne	*C. crispatula*	Aquatic	1.78
	C. lingua	Aquatic	1.80
	C. nevilli	Aquatic	1.90
Dieffenbachia	*D. seguine*	Terrestrial	24.66
Dracunculus	*D. canariensis*	Terrestrial	7.78
	D. vulgaris	Terrestrial	13.70
Epipremnum	*E. aureum*	Nomadic vine	8.08
	E. aureum	Nomadic vine	9.40
Homalomena	*rubescens*	Terrestrial	18.28
Lemna	*L. aequinoctialis*	Aquatic	0.86
	L. gibba	Aquatic	1.00
	L. japonica	Aquatic	0.88
	L. minor	Aquatic	2.91
	L. minor	Aquatic	1.13
	L. obscura	Aquatic	1.00
	L. trisulca	Aquatic	0.92
	L. valdiviana	Aquatic	0.66
Monstera	*M. deliciosa*	Nomadic vine	8.41
	M. obliqua	Nomadic vine	18.01
Orontium	*O. aquaticum*	Aquatic (helophyte)	30.00
Philodendron	*P. erubescens*	Terrestrial	10.56
	P. melanochrysum	Nomadic vine	2.40
	P. pinnatafidum	Terrestrial	4.88
	P. squamiferum	Nomadic vine	9.30
Pinellia	*P. pedatisecta*	Terrestrial	2.44
	P. pedatisecta	Terrestrial	2.79
	P. tripartita	Terrestrial	7.05
Pistia	*P. stratiotes*	Aquatic	0.70
Remusatia	*R. vivipara*	Facultative epiphyte	4.26
Rhaphidophora	*S. montana*	Nomadic vine	20.06
	S. peepla	Nomadic vine	18.34
Scindapsus	*S. pictus*	Nomadic vine	23.51
Spathicarpa	*S. hastifolia*	Terrestrial	18.92
Spathiphyllum	*S.* sp. cv. »Cupido«	Terrestrial	14.10

	S. sp. cv. »Macho«	Terrestrial	20.10
	S. sp. cv. »Mauna Loa«	Terrestrial	7.10
Spirodela	*S. polyrhiza*	Aquatic	0.60
	S. punctata	Aquatic	0.74
	S. punctata	Aquatic	0.82
Symplocarpus	*S. foetidus*	Terrestrial	4.80
Syngonium	*S. angustatum*	Nomadic vine	9.45
	S. podophyllum	Nomadic vine	9.71
Thaumatophyllum	*T. bipinnatifidum*	Nomadic vine	9.99
Typhonium	*T. flagelliforme*	Terrestrial	10.34
	T. trilobatum	Terrestrial	13.17
Wolffia	*W. angusta*	Aquatic	3.40
	W. arrhiza	Aquatic	3.27
	W. australiana	Aquatic	0.76
	W. borealis	Aquatic	1.82
	W. brasiliensis	Aquatic	1.58
	W. columbiana	Aquatic	1.78
	W. cylindracea	Aquatic	2.20
	W. elongata	Aquatic	1.74
	W. globosa	Aquatic	2.64
	W. microscopica	Aquatic	3.40
	W. neglecta	Aquatic	2.40
Wolffiella	*W. gladiata*	Aquatic	1.28
	W. hyalina	Aquatic	1.98
	W. lingulata	Aquatic	1.34
	W. oblonga	Aquatic	1.52
Xanthosoma	*X. sagittifolium*	Terrestrial	17.57
	X. sagittifolium	Terrestrial	4.60
Zamioculcas	*Z. zamiifolia*	Terrestrial	48.10
Zantedeschia	*Z. aethiopica*	Terrestrial	4.60
	Z. albomaculata	Terrestrial	4.60

Table S2. List of aroid species with harmonized names based on current taxonomy [18,31,32].

Royal Botanic Gardens Kew database	Synonym after current taxonomy
Alocasia hilobeauty	*Caladium praetermissum*
Alocasia lowii var. grandis	*Alocasia longiloba* var. grandis
Alocasia x amazonica	*Alocasia sanderiana*
Amorphophallus dubius	*Amorphophallus paeoniifolius*
Amorphophallus laxiflorus	*Amorphophallus gallaensis*
Amorphophallus rivieri	*Amorphophallus konjac*
Amorphophallus sutepensis	*Amorphophallus krausei*
Arum elongatum	*Arum orientale*
Colocasia antiquorum	*Colocasia esculenta*
Dieffenbachia picta	*Dieffenbachia seguine*
Landoltia punctata	*Spirodela punctata*
Philodendron andreanum	*Philodendron melanochrysum*
Philodendron selloum	*Thaumatophyllum bipinnatifidum*
Scindapsus aureus	*Epipremnum aureum*
Spathicarpa sagittifolia	*Spathicarpa hastifolia*
Syngonium albo-lineatum	*Syngonium angustatum*
Typhonium cuspidatum	*Typhonium flagelliforme*
Xanthosoma violaceum	*Xanthosoma sagittifolium*
